# Cholesterol secosterol aldehyde adduction and aggregation of Cu,Zn-superoxide dismutase: Potential implications in ALS

**DOI:** 10.1016/j.redox.2018.08.007

**Published:** 2018-08-16

**Authors:** Lucas S. Dantas, Adriano B. Chaves-Filho, Fernando R. Coelho, Thiago C. Genaro-Mattos, Keri A. Tallman, Ned A. Porter, Ohara Augusto, Sayuri Miyamoto

**Affiliations:** a Departamento de Bioquímica, Instituto de Química, Universidade de São Paulo, São Paulo, SP, Brazil; b Department of Chemistry, Vanderbilt Institute of Chemical Biology and Vanderbilt Kennedy Center for Research on Human Development, Vanderbilt University, Nashville, TN, United States

**Keywords:** a-Ch, alkynyl-cholesterol, a-HNE, alkynyl-4-hydroxynonenal, a-Seco A, alkynyl-Secosterol A, a-Seco B, alkynyl-Secosterol B, ALS, amyotrophic lateral sclerosis, DHA, Docosahexaenoic acid, HCCA, alphacyano-4-hydroxycinnamic acid, HPLC-UV, high-performance liquid chromatography ultraviolet detection, MALDI-TOF, matrix assisted laser desorption ionization, time-of-flight, PBH, 1-pyrenebutiric hydrazine, SDS−PAGE, sodium dodecyl sulfate, polyacrylamide gel electrophoresis, SEC, size exclusion chromatography, Seco-A, 3β-hydroxy-5-oxo-5,6-secocholestan-6-al, Seco-B, 3β-hydroxy-5β-hydroxy-B-norcholestane-6β-carboxyaldehyde, SOD1, cooper, zinc-superoxide dismutase, TEM, transmission electronic microscopy, ThT, thioflavin T, Amyotrophic Lateral Sclerosis, Secosterol aldehydes, Neurodegenerative diseases, Superoxide dismutase, Protein aggregation

## Abstract

Amyotrophic lateral sclerosis (ALS) is a neurodegenerative disorder characterized by degeneration of upper and lower motor neurons. While the fundamental causes of the disease are still unclear, the accumulation of Cu,Zn-superoxide dismutase (SOD1) immunoreactive aggregates is associated with familial ALS cases. Cholesterol 5,6-secosterol aldehydes (Seco A and Seco B) are reported to contribute to neurodegenerative disease pathology by inducing protein modification and aggregation. Here we have investigated the presence of secosterol aldehydes in ALS SOD1-G93A rats and their capacity to induce SOD1 aggregation. Secosterol aldehydes were analyzed in blood plasma, spinal cord and motor cortex of ALS rats at the pre-symptomatic and symptomatic stages. Seco B was significantly increased in plasma of symptomatic ALS rats compared to pre-symptomatic animals, suggesting an association with disease progression. *In vitro* experiments showed that both Seco A and Seco B induce the formation of high molecular weight (HMW) SOD1 aggregates with amorphous morphology. SOD1 adduction to ω-alkynyl-secosterols analyzed by click assay showed that modified proteins are only detected in the HMW region, indicating that secosterol adduction generates species highly prone to aggregate. Of note, SOD1-secosterol adducts containing up to five secosterol molecules were confirmed by MALDI-TOF analysis. Interestingly, mass spectrometry sequencing of SOD1 aggregates revealed preferential secosterol adduction to Lys residues located at the electrostatic loop (Lys 122, 128 and 136) and nearby the dimer interface (Lys 3 and 9). Altogether, our results show that secosterol aldehydes are increased in plasma of symptomatic ALS rats and represent a class of aldehydes that can potentially modify SOD1 enhancing its propensity to aggregate.

## Highlights

• Secosterol aldehydes are ubiquitously present in motor cortex and spinal cord;
• These aldehydes modify SOD1 in vitro, producing high molecular weight aggregates;
• Lys residues at electrostatic loop and nearby the dimer interface are mainly modified;
• Secosterol adduction increases protein hydrophobicity and its propensity to aggregate.

## Graphical abstract

**Figure:**
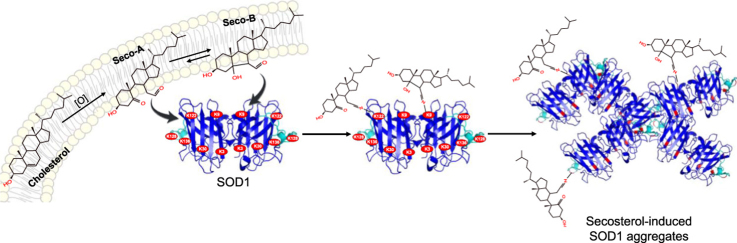

## 1 Introduction

**Fig. 1:**
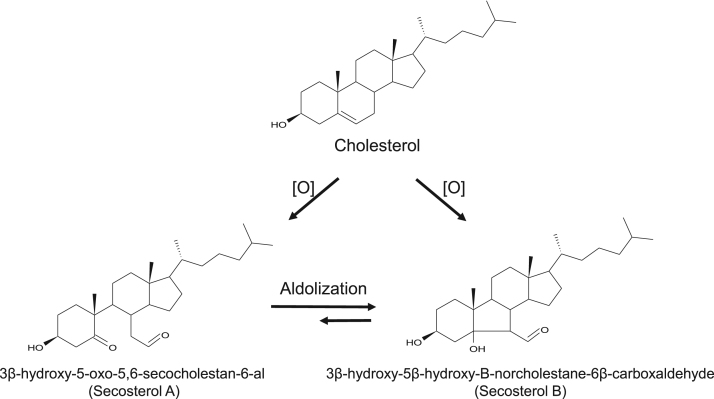
Chemical structures of cholesterol 5,6-secosterol aldehydes (Secosterol A, Seco A; and Secosterol B, Seco B). These electrophilic oxysterol derivatives are generated by the oxidation of cholesterol induced by singlet molecular oxygen, ozone and free radicals.

Cholesterol is a neutral lipid found in the membranes of all mammalian cells. The central nervous system is particularly rich in cholesterol, presenting a concentration near to 20 mg/g in the brain and 40 mg/g in spinal cord, which represents about 23% of the total sterol present in the body [1]. Cholesterol, like all other unsaturated lipids, is susceptible to oxidation, giving rise to a variety of oxidized derivatives, collectively known as oxysterols [2], [3], [4]. This lipid can be oxidized by enzymatic and non-enzymatic mechanisms. Non-enzymatically, cholesterol can be oxidized by singlet molecular oxygen (^1^O_2_), ozone (O_3_), and free-radicals [5], [6], [7], [8], [9], [10], [11]. Among the major products of cholesterol oxidation are the isomeric hydroperoxides (7α-OOH, 7β-OOH, 5α-OOH, 6α-OOH, 6β-OOH), epoxides, and aldehydes [6], [7], [8], [11]. Attention has been particularly given to cholesterol 5,6-secosterol aldehydes (Seco A and Seco B) (Fig. 1), two electrophilic oxysterols known to be formed from cholesterol oxidation intermediates formed by O_3_ [9], [10] and ^1^O_2_ [8], [11]. More recently, Zielinsk and Pratt demonstrated that these aldehydes can also arise from free radical mediated oxidation of cholesterol [12], suggesting that their formation does not require the presence of high energy oxygen intermediates (i.e. O_3_ or ^1^O_2_).

Secosterol aldehydes have been detected in samples of atherosclerotic tissue and human brain as important intermediates in the pathogenesis of cardiovascular [13], [14] and neurodegenerative diseases [15], [16], [17], [18]. These electrophilic oxysterol derivatives can modify specific proteins in the brain, such as β-amyloid peptide [15], [16], [19], [20], α-synuclein [17] and bovine myelin basic protein [18], leading to the formation of protein aggregates. In addition, secosterol aldehydes may also modify other proteins such as apoB-100 [13], p53 [21], NO synthase [14] and cytochrome c [22], resulting in structural change and function loss.

SOD1 is a soluble antioxidant enzyme present in the cytosol, nucleus, peroxisomes and mitochondrial intermembrane space of eukaryotic cells [23], [24]. SOD1, as well as its other isoforms, is responsible for the catalytic dismutation of the superoxide radical anion to hydrogen peroxide and molecular oxygen. Mutations of the SOD1 gene are linked to some cases of Amyotrophic Lateral Sclerosis (ALS) [25]. ALS is a fatal neurodegenerative disease manifested in adults with an incidence of 1–2/100,000 in most populations. Typical ALS develops between 50 and 60 years of age, with progressive neuromuscular failure caused by motor neurons degeneration in the brain and spinal cord causing denervation and consequent muscle atrophy. The etiology of the disease is still unknown. Whereas the majority of diagnosed cases have been characterized of sporadic origin (sALS), it is estimated that 5–10% of the cases are genetic, classified as familiar ALS (fALS) [26]. In 1993, Deng and colleagues identified the first mutated gene in ALS, responsible for approximately 25% of autosomal dominant fALS cases. This gene encodes the enzyme SOD1 which is located on chromosome 21 [25].

Although more than 150 mutations have been identified in the SOD gene, the molecular mechanisms of selective degeneration of motor neurons in SOD1 fALS mutants are still unclear. The mutation results in a gain of toxic function, which has suggested to cause pro-oxidant effects and/or formation of cytotoxic SOD1 aggregates [27], [28]. The structural events that lead to the formation of high molecular weight SOD1 oligomers have been extensively debated [27]. These events likely involve intermolecular covalent disulfide bonds at cysteines 6 and 111, and non-covalent interactions between beta sheets, forming the ß-amyloid-like structures [29]. A number of factors can enhance protein aggregation, including zinc-deficiency [30], [31], protein oxidative modifications [28] and the interaction with membrane lipids [32], [33], [34]. Yet, the interaction between SOD1 and lipids has been underexplored as compared to key proteins involved in other diseases [35], [36], [37].

Considering the high abundance of cholesterol in brain and spinal cord [1] and the electrophilic nature of its oxidation products, we sought to investigate the presence of secosterol aldehydes in neural tissues and plasma from an ALS rat model and also to evaluate their potential to induce SOD1 aggregation. Here we found higher amounts of Seco B in blood plasma of ALS rat model (SOD1-G93A rats) at symptomatic (~130 days) compared to pre-symptomatic (~70 days) stage. More importantly, secosterol aldehydes dramatically enhanced SOD1 aggregation through a mechanism involving covalent modifications of Lys residues mainly at the electrostatic loop (Lys 122, 128 and 136) and at the dimer interface (Lys 3 and 9).

## 2 Materials and methods

### 2.1 Materials

Secosterol A (3β-hydroxy-5-oxo-5,6-secocholestan-6-al) was synthesized by the ozonization of cholesterol and purified as described by Wang and colleagues [38]. Secosterol B (3β-hydroxy-5β-hydroxy-B-norcholestane-6β-carboxaldehyde) was synthesized by the photooxidation of cholesterol and purified as described by Uemi and colleagues [8]. SOD1 was expressed in *Escherichia coli*, purified and its apo form prepared as previously described [39]. Alkynyl lipids were synthesized as previously described [40]. Unless otherwise stated all chemicals were of the highest analytical grade and were purchased from Sigma, Merck or Fisher.

### 2.2 ALS rat model

Sprague Dawley hemizygous male rats overexpressing multiple copies (~8 copies) of G93A mutant human copper,zinc-superoxide dismutase (hSOD1^G93A^) were obtained from Taconic (Germantown, NY) [41]. Genotyping was performed at approximately 20 days of age using ear DNA to identify transgenic progeny expressing the hSOD1^G93A^ transgene. Rats were housed under controlled environment with a 12 h light-dark cycle with access to food and water ad libitum. The development of characteristic symptoms of the disease was accompanied by evaluation of animal's body weight and loss of limbs movement. The animals were considered symptomatic when they showed a ~20% loss of their maximum body weight accompanied by atrophy/paralysis of the limbs. Pre-sympotomatic (~70 days, n = 9) and symptomatic animals (132 ± 12 days, n = 10) were sacrificed together with their aged matched controls. Rats were fasted for 6 h and deeply anesthetized with ketamine (100 mg/kg) and xylazine (2 mg/kg). Blood was collected from the right atrium of the heart into a heparin containing tube and centrifuged to separate the plasma. Motor cortex and spinal cord were collected and stored at −80 °C until use. All animal procedures were approved by University of Sao Paulo - Chemistry Institute's Animal Care and Use Committee under the protocol number 14/2013.

### 2.3 Quantification of Cholesterol Secosterol Aldehydes

Spinal cord and motor cortex were weighed to prepare a 200 mg/mL (w/v) homogenate. Tissues were homogenized in cold phosphate buffer solution (10 mM, pH 7.4 containing 0.1 mM desferoxamine mesylate) using a Potter-Elvehjem homogenizer. Homogenates were centrifuged for 2.000 g for 5 min at 4 °C and the upper phase was collected and stored at −80 °C. For the analysis, an aliquot of the plasma and homogenates were subjected to lipid extraction according to the method of Bligh & Dyer [42]. The analysis of secosterol aldehydes was performed using the method previously described by our group, in which aldehydes were derivatized with a fluorescent probe 1-pyrenebutiric hydrazine (PBH) [43]. To increase the precision secosterol aldehyde quantification we used sitosterol-derived aldehyde (Sito-Ald) as an internal standard. Details of the method are described in the supporting information. Cholesterol concentration was determined by HPLC-UV detection at 205 nm using a C18 column (Kinetex, 50 ×4.6 mm 2.6 µm, Phenomenex) eluted isocratically with 95% methanol and 5% water. A 5 µL aliquot was injected for the analysis and cholesterol levels were calculated according to a calibration curve. Results are shown as mean ± S.D. and differences were determined by ANOVA with Tukey post-test. A *p* value of 0.05 or less was used as the criterion for statistical significance.

### 2.4 SOD1 aggregation experiments

SOD1 WT (10 µM) in apo and holo forms were incubated in 50 mM phosphate buffer pH 7.4 containing 150 mM NaCl and 100 µM DTPA for 24 h at 37 °C in the presence of 250 µM cholesterol, cholesterol hydroperoxides, Seco A or Seco B. Docosahexaenoic acid (DHA, 250 uM) was used as a positive control [33]. For detection of SOD1 oligomers, SDS-PAGE was performed under reducing (+β-mercaptoethanol) and non-reducing (-β-mercaptoethanol) conditions in a 12% polyacrylamide gel. Aliquots of the samples (20 µL) were incubated in sample buffer (62 mM Tris-HCl, pH 6.8 containing 10% glycerol, 2% SDS, 0.01% bromophenol blue) in the absence and presence of β-mercaptoethanol (200 mM) for 5 min at 95 °C and then applied on the gel. Silver nitrate was applied for gel staining. Size exclusion chromatography (SEC) was performed using the column BioSep-SEC-S3000 (300 ×7.8 mm, Phenomenex, USA). Each sample was eluted with 50 mM phosphate buffer, pH 7.4 containing 150 mM NaCl. Fluorescence detector conditions were: excitation wavelength at 280 nm and emission at 340 nm.

### 2.5 Effects of pH and secosterol aldehyde concentration on SOD1 aggregation

For pH evaluation, solutions of 50 mM phosphate buffer containing 150 mM NaCl and 100 µM DTPA were made in different pHs: 4.7, 5.5, 6.2, 7.4, 8.4 and 10. Apo-SOD1 (10 µM) was incubated with Seco A or Seco B (250 µM) at all pHs for 24 h at 37 °C. To evaluate the effect of concentration, incubations of apo-SOD1 (10 µM) were performed in increasing concentrations from 10 to 250 µM cholesterol aldehydes for 24 h at 37 °C. Aggregate analysis was performed by SEC.

### 2.6 Analysis of SOD1 aggregates morphology

The increase of hydrophobicity at the protein surface was monitored by following the increase in fluorescence of thioflavin T (ThT). After 24 h of incubation, 10 µM ThT was added to the samples and after 15 min at 37 °C, fluorescence was recorded on a plate reader (TECAN, Switzerland) with excitation at 440 nm and emission at 485 nm. To evaluate whether SOD1 aggregates were formed by amyloid fibers, congo red (CR) assay was performed. This reagent suffers a red shift on its absorbance peak after binding to amyloid fibers. For the analysis, the incubations were mixed with 6 μM CR in 5 mM phosphate buffer pH 7.4. A positive control was an incubation of α-synuclein with seco A, which has been known to form amyloid fibrils [17]. Spectrophotometric analyses were carried out in a Varian model Cary 50 Bio spectrophotometer. Aggregate morphology was also analyzed by transmission electron microscopy (TEM) as described previously [44].

### 2.7 Click chemistry assay

Apo-SOD1 (10 µM) was incubated with 1 mM alkynyl lipids (a-HNE, a-Ch, a-Seco A and a-Seco B) in 50 mM phosphate buffer pH 8.4 containing 150 mM NaCl and 100 µM DTPA for 24 h at 37 °C. The samples were then reduced with 5 mM sodium borohydride for 1 h at room temperature to stabilize possible adducts, and finally neutralized with 10% HCl. The following click reagents were added to each of the samples: azido-biotin reagent (0.2 mM), tris(3-hydroxypropyltriazolylmethyl)amine (THPTA) ligand (0.2 mM), copper sulfate (1 mM), and sodium ascorbate (1 mM), and the samples were vortexed and allowed to stir for 2 h in the dark at room temperature. Biotinylated samples were resolved by SDS-PAGE as described above. The protein was transferred electrophoretically to a polyvinylidene fluoride membrane (Life Technologies, Grand Island, NY) and probed with streptavidin conjugated with the Alexa Fluor 680 fluorophore (Life Technologies). Biotinylated proteins were visualized using the Odyssey Infrared Imaging System and Odyssey software according to the manufacturer (Licor, Lincoln, NE).

### 2.8 MALDI-TOF analysis

Apo-SOD1 (10 µM) was incubated with 1 mM cholesterol, Seco A or Seco B in 50 mM phosphate buffer pH 8.4 containing 150 mM NaCl and 100 µM DTPA for 24 h at 37 °C. The samples were then reduced with 5 mM sodium borohydride for 1 h at room temperature to stabilize possible adducts. After that, the protein was denatured at 95 °C for 5 min and the aggregates were reduced with 1 M dithiothreitol (DTT) for 1 h at room temperature. Methanol (750 µL) was added to the samples, which were left on ice for 20 min to precipitate SOD1. After centrifugation at 10,000 rpm for 5 min, the precipitate was suspended in 100 µL of 0.1% trifluoroacetic acid for MALDI-TOF analysis. Samples were mixed in a 1:4 (v/v) ratio with a saturated solution of α-cyano-4-hydroxycinnamic acid (HCCA) in 50% acetonitrile/0.1% aqueous trifluoroacetic acid (1:1, v/v). Approximately 1 µL of the resulting mixture was spotted onto a MALDI target and analyzed by MALDI-TOF MS. The analyses were performed in the linear, positive ion mode in an UltrafleXtreme spectrometer (Bruker Daltonics, Germany) using an acceleration voltage of 25 kV. The resulting spectra were analyzed by flexAnalysis software (Bruker Daltonics, Germany).

### 2.9 Enzymatic digestion of SOD1 and peptide analysis

SOD1 samples incubated in the presence of cholesterol aldehydes were first reduced with 5 mM sodium borohydride for 1 h at room temperature. After that, cysteine residues were reduced with 5 mM DTT (dithiotreitol) and alkylated with 15 mM iodoacetamide. Then, samples were digested for 18 h with proteomic grade trypsin (Promega) in a 1:100 (w/w) ratio at 37 °C with aid of RapiGest SF Surfactant (Waters). The resulting peptides were analyzed by LC-MS/MS using a nanoAcquity UPLC system (Waters, United States) coupled to a TripleTOF 6600 mass spectrometer (Sciex, United States). Analysis was conducted under trap and elute mode using a nanoAcquity UPLC-Symmetry C18 trap column (20 mm x 180 µm; 5 µm) and separation column (75 µm x 150 mm; 3,5 µm). Trapping was done at 10 µL/min with 1% of solvent B. Peptides were separated with mobile phase A (0.1% formic acid in water) and B (0.1% formic acid in acetonitrile) at a flow rate of 0.4 µL/min using the following gradient: 1–35% B from 0 to 60 min; 35–90% B from 60 to 61 min; isocratic elution with 90% B from 61 to 73 min; 90–1% B from 73 to 74 min. Nano-electrospray ion source was operated at 2.4 kV (ion spray voltage floating, ISVF), curtain gas 20, interface heater (IHT) 120, ion source gas 1 (GS1) 3, ion source gas 2 (GS2) zero, declustering potential (DP) 80 V. TOFMS and MS/MS data were acquired using information-dependent acquisition (IDA) mode. For IDA parameters, a 100 ms survey scan in the *m/z* range of 300–2000 was followed by 25 MS/MS ions in the *m/z* range of 100–2000 acquired with an accumulation time of 50 ms (total cycle time 1.4 s). Switch criteria included, intensity greater than 150 counts and charge state 2–5. Former target ions were excluded for 20 s. Software used for acquisition, data processing, and quantification were Analyst TF 1.7, PeakView 2.1 and MultiQuant 3.0, respectively. For the analysis of protein modification, MASCOT software (Matrix Science Ltd., London, United Kingdom) was used with mass tolerance of 10 ppm for MS experiments and 0.05 Da for MS/MS experiments. Search configuration was set to have variable modifications of + 57.0214 Da for carbamidomethyl to Cys, + 400.3341 Da for Michael addition of secosterols to Cys, Lys and His, and + 402.3497 Da for Schiff base adduction of secosterols to Lys.

## 3 Results

### 3.1 Secosterol aldehyde detection in blood plasma, motor cortex and spinal cord

**Fig. 2:**
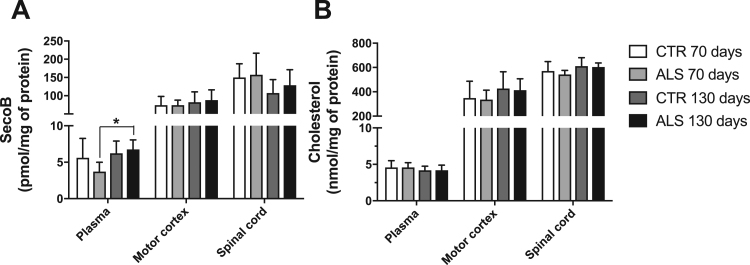
Quantification of cholesterol secosterol aldehydes in plasma, motor cortex and spinal cord obtained from ALS rat model (SOD1-G93A) at the pre-symptomatic (~70 days, n = 9) and symptomatic stage (~130 days, n = 10). ALS rats over-expressing SOD1-G93A mutant protein were sacrificed together with the corresponding age-matched WT controls (CTR). Lipids were extracted to analyze secosterol aldehydes (Seco A and Seco B) content as described in the Experimental Procedures and Supporting Information. Ony Seco B was detected. Seco B concentration was normalized by protein concentration in blood plasma (A), motor cortex (B) and spinal cord (C). Values correspond to the mean ± SD; *p < 0.05 (ANOVA, Tukey post-test).

First, we sought to investigate the presence of secosterol aldehydes in nervous tissues and blood plasma of an ALS rat model (SOD1-G93A rats). For this purpose, we collected motor cortex, spinal cord and plasma from rats at pre-symptomatic (~70 days) and symptomatic stages (~130 days). We detected significant amounts of Seco B but no detectable levels of Seco A. This is consistent with studies showing that Seco A is converted to Seco B within 24 h in aqueous phosphate buffer solution [9], [13], [17]. Seco A aldolization to Seco B can be catalyzed under in vivo conditions and Seco B seems to be the major product detected in biological tissues [9]. Thus, Seco B detected in our samples can be considered as the sum of Seco A and Seco B. Importantly, secosterol aldehydes were significantly higher in blood plasma of symptomatic compared to pre-symptomatic ALS animals (Fig. 2A). Significant differences were not found in motor cortex and spinal cord. However, a trend towards an increase in secosterol aldehyde was observed in the motor cortex of symptomatic animals.

### 3.2 Secosterol aldehydes induce SOD1 aggregation in vitro

**Fig. 3:**
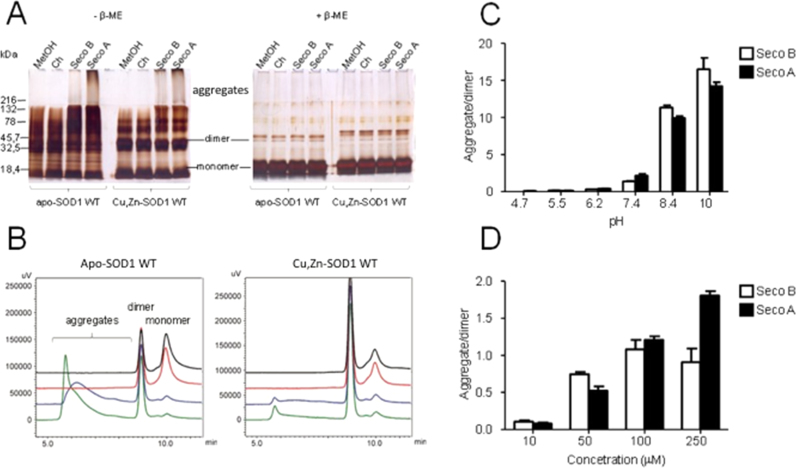
**SOD1 oligomerization induced by secosterol aldehydes (Seco A and Seco B).** Incubations contained 10 μM SOD1 and 250 μM cholesterol, Seco A or Seco B in 50 mM phosphate buffer at pH 7.4. After incubation at 37 °C for 24 h, SOD1 oligomerization was analyzed by SDS-PAGE (A) and size-exclusion chromatography, SEC (B). Large aggregates (>200 kDa) of both apo- and Cu, Zn-SOD1 (holo form) were observed in the SDS-PAGE analysis without β-ME. SEC analysis of apo-SOD1 (left panel) and Cu, Zn-SOD1 (right panel) incubated with control solvent (black line), cholesterol (red line), Seco B (blue line) and Seco A (green line). (C) pH dependent formation of apo-SOD1 WT aggregates. (D) Secosterol aldehydes increased apo-SOD1 WT aggregation in a concentration dependent manner.

Next, we investigated the ability of secosterol aldehydes to promote SOD1 aggregation. Interestingly, Seco A and Seco B dramatically enhanced the formation of high molecular weight SOD1 aggregates (> 100 kDa), which appeared as a smear at the top of non-reducing SDS-PAGE (Fig. 3A). The same trend was not observed in incubations containing cholesterol (Ch), cholesterol hydroperoxide (ChOOH) or solvent (10% methanol or isopropanol), although the later showed oligomerization to some extent (Fig. S4). The aggregating effect was observed for both apo and holo-SOD1 forms, pointing to a highly destabilizing nature of the modifications promoted by secosterol aldehydes. Notably, under reducing conditions, all high molecular weight bands disappeared, showing that SOD1 aggregates are mainly maintained by intermolecular disulfide crosslinking (Fig. 3A).

Next, we confirmed the formation of SOD1 aggregates by size exclusion chromatography (SEC) analysis. As expected, high molecular weight aggregates were only observed when SOD1 was incubated with secosterol aldehydes, but not with intact cholesterol or solvent. Interestingly, aggregation preferentially occurred with consumption of SOD1 monomer, consistent with previous studies showing high propensity of SOD1 monomers to aggregate [45].

SOD1 aggregation induced by secosterol aldehydes was pH dependent (Fig. 3C). Aggregation was greatly enhanced at neutral to alkaline pH but did not occur at acidic conditions (pH<7). We also tested different concentrations of the aldehyde. Aggregation was observed at concentrations as low as 10 µM (aldehyde: protein, 1:1 M ratio) and increased almost linearly up to 250 µM reaching a plateau with Seco B at 100 µM (Fig. 3D). Time-dependent analysis showed a rather fast aggregation kinetics reaching a plateau after 12–24 h, with Seco A and B, respectively (Fig. S5). Moreover, Seco A displayed higher ability to induce aggregation compared to Seco B at 250 µM (pH 7.4).

### 3.3 Aggregates formed by secosterol aldehydes have amorphous nature

**Fig. 4:**
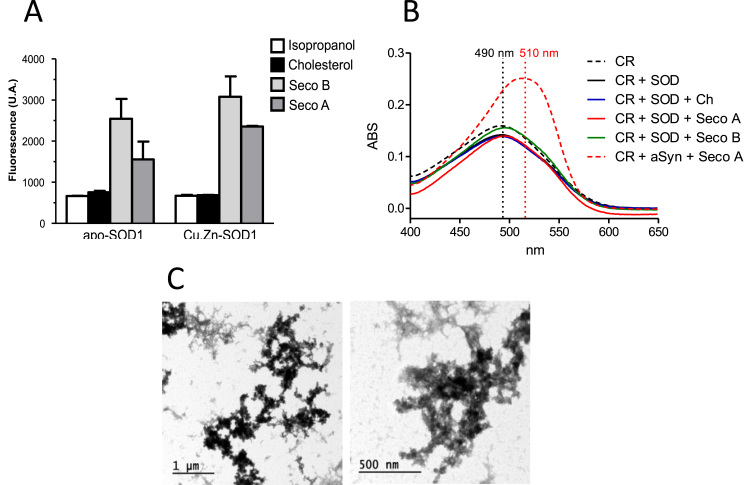
**Morphology of SOD1 aggregates formed in the presence of secosterol aldehydes.** (A) Effect of secosterol aldehydes on the exposition of hydrophobic residues of SOD1 analyzed by thioflavin T fluorescence assay. (B) Absorption spectrum of congo red obtained for incubation containing SOD1 and cholesterol or secosterol aldehydes (Seco A or Seco B). As positive control for fibrillar aggregates we used α-synuclein incubated with Seco A (dashed red line), which showed the typical spectral red shift. (C) Transmission electronic microscopy (TEM) images of the apo-SOD1 WT incubated with Seco B showing granules with amorphous morphology.

Alterations in protein conformation leading to exposure of protein hydrophobic residues can trigger the formation of β-amyloid type aggregates [29], [46]. To check the morphology of SOD1 aggregates we first conducted dye binding experiments with thioflavin T (ThT). Experiments with apo and holo forms of SOD1 treated with aldehydes showed a 2–3 fold enhancement of ThT fluorescence after 24 h incubation, indicating the formation of amyloid-like structures (Fig. 4A). However, a Congo Red (CR) binding experiment did not show changes in the absorbance, nor the characteristic red spectral shift typically observed for amyloid fibrils (Fig. 4B, alpha-synuclein was used as positive control for amyloid fibrils). Thus, to clarify the morphological nature of SOD1 aggregates we also performed TEM analysis. In agreement with CR results, TEM images showed the presence of aggregates displaying amorphous morphology (Fig. 4C).

### 3.4 SOD1 aggregates are bound to secosterol aldehydes

**Fig. 5:**
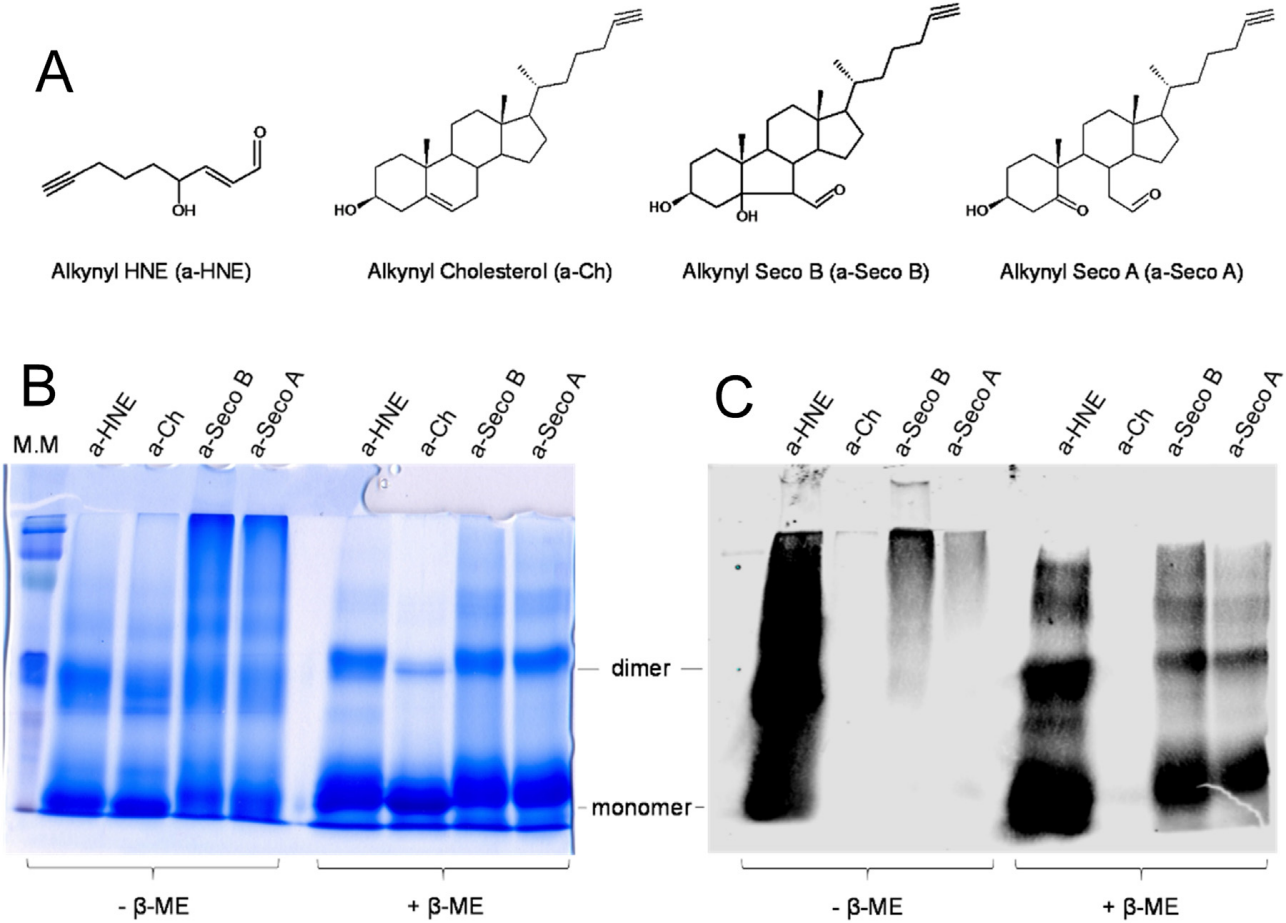
**Analysis of apo-SOD1 adduction to alkynyl analogues of HNE or secosterol aldehydes by click chemistry.** (A) ω-alkynyl lipid structures: alkynyl cholesterol (a-Ch); alkynyl Seco B (a-Seco B); and alkynyl Seco A (a-Seco A). (B) SDS-PAGE of the apo-SOD1 WT (10 μM) incubated with alkynyl-lipids (250 μM) analyzed under non-reducing (-β-ME) and reducing conditions (+β-ME) and stained with Coomassie-blue. a-Seco A and Seco B induced the formation of large aggregates, while a-HNE did not. (C) Detection of SOD1 adduction to alkynyl eletrophiles by click chemistry. SOD1 modified by alkynyl lipids were labeled with biotin and stained with streptavidin conjugated with the Alexa Fluor 680 fluorophore. Intensive staining was found for a-HNE, while for a-Seco A and a-Seco B, staining was concentrated in the large aggregate region.

As a way to detect SOD1-secosterol adducts we used clickable alkynyl derivatives [40]. SOD1 was incubated in the presence of alkynyl tagged-Cholesterol (a-Ch, Control), -Seco A (a-Seco A), -Seco B (a-Seco B) and -4-hydroxy-2-nonenal (a-HNE) (Fig. 5A). The latter is a highly reactive short chain lipid-derived eletrophile that was used for comparative purposes. In this assay, alkynyl lipids attached to proteins can be identified with the azido-biotin reagent via Huisgen-Sharpless cycloaddition and subsequently detected with a fluorescent streptavidin conjugate [40] (Fig. S6). Interestingly, incubations of apo-SOD1 with alkynyl tagged secosterol aldehydes (a-Seco A and a-Seco B) showed intense labeling only in the high molecular weight (HMW) region at non reducing gels with no detectable adducted monomers or dimers (Fig. 5C). Labeled monomeric and dimeric SOD1 forms became visible only after reduction with beta-mercaptoethanol (Fig. 5C). Conversely, a-HNE showed intense labeling spread over almost the entire gel (Fig. 5C), reflecting its high reactivity with the protein. Of note, a-HNE adduction did not gave HMW aggregates as can be noted by the lack of the smear at the top of non-reducing gel stained with Coomassie-blue (Fig. 5B). This result implies that modifications induced by secosterol aldehydes convert the protein to species highly prone to undergo aggregation whereas a-HNE adduction induces less aggregation.

**Fig. 6:**
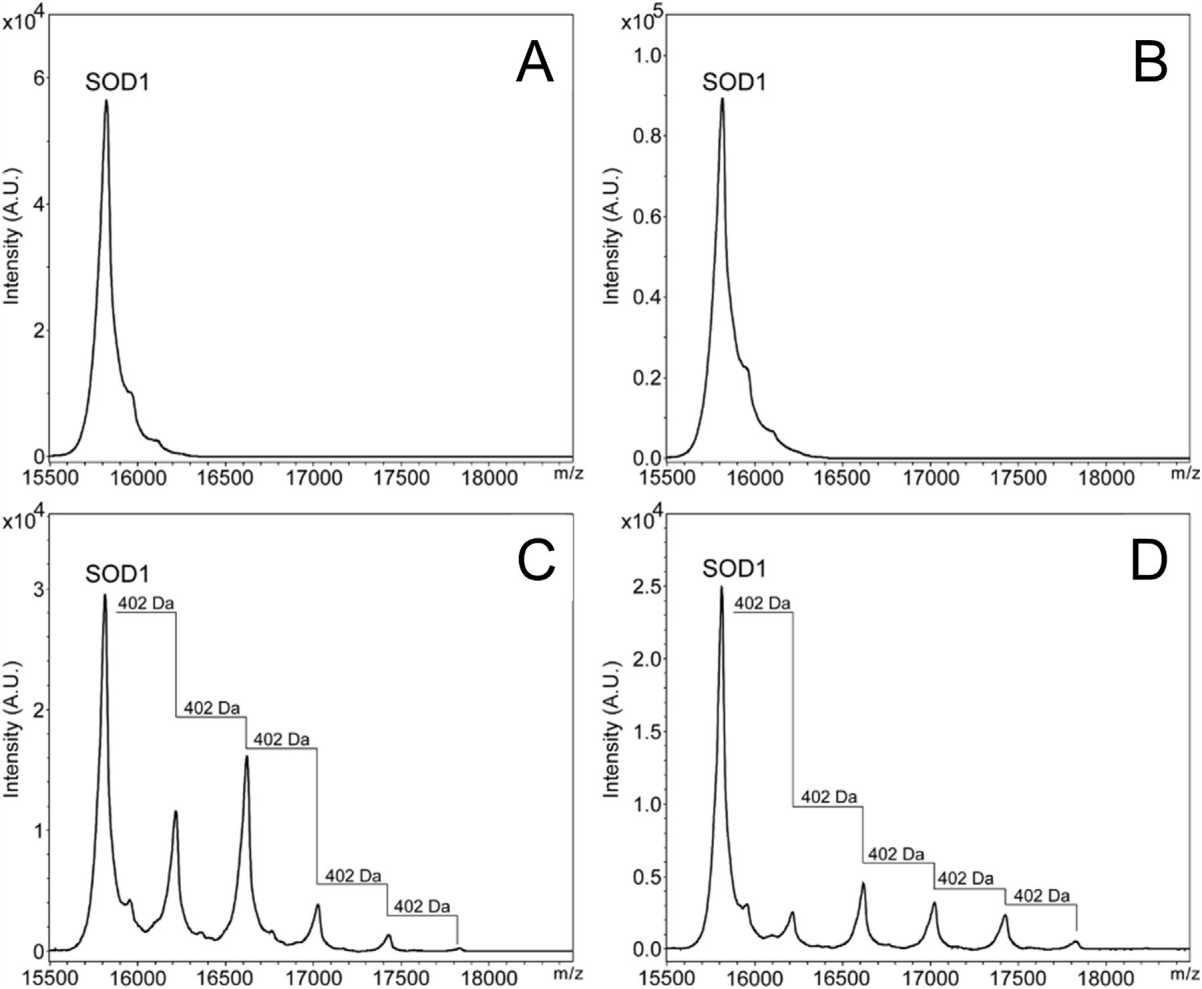
**SOD1-secosterol covalent adducts detected by MALDI-TOFMS.** Covalent adducts were analyzed after 24 h incubation of the protein in the absence (A) or presence of cholesterol (B), cholesterol aldehydes Seco B (C) or Seco A (D). After incubation, Schiff base adducts were reduced by NaBH_4_ and SOD1 aggregates were disrupted by the addition of DTT, alkylated with IAM and then analyzed by MALDI-TOFMS.

To characterize the secosterol-SOD1 adducts, aggregated samples were reduced by DTT and analyzed by MALDI-TOF mass spectrometry. The MS spectrum showed the presence of up to five modifications in the protein, differing from the intact protein by the addition of 402 Da (Fig. 6C and D). Such mass difference is consistent with the formation of Schiff base adducts between the secosterol aldehyde (M=418 Da) and basic amino acid residues, such as lysine, after a reduction step with sodium borohydride.

Taken together, these results suggest that secosterol aldehydes (Seco A and Seco B) produce covalently modified SOD1-adducts that are much more prone to undergo olygomerization into high molecular weight aggregates when compared to 4-HNE.

### 3.5 SOD1 is modified mainly at lysine residues

To identify the sites of covalent adduction by secosterol aldehydes, SOD1 aggregates were digested by trypsin and submitted to nano-LC-MS/MS. Protein sequencing by Mascot software gave a coverage greater than 99%. Among modified residues we found the carbamidomethylated cysteine residues at Cys6, Cys57, Cys111 and Cys146. Moreover, it was possible to identify six secosterol Schiff-base adducts (peptides with mass shift of 402.3497 Da) on six Lys residues, namely: Lys3, Lys9, Lys30, Lys122, Lys128 and Lys136.

**Fig. 7:**
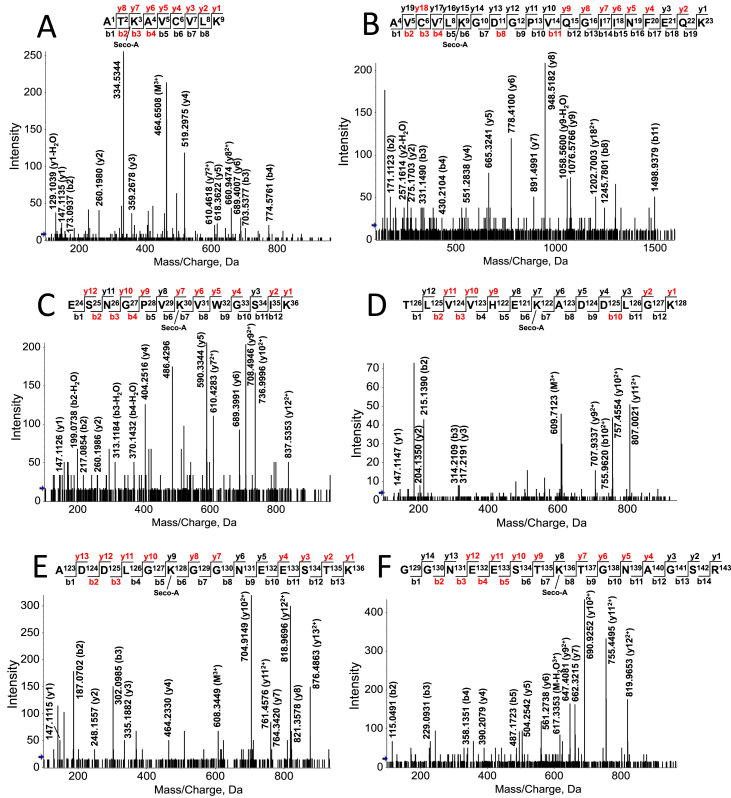
**nano-LC-MS/MS sequencing of secosterol modified peptides derived from the tryptic digestion of SOD1 incubated with Seco A.** Incubations contained 10 μM SOD1 with 250 μM Seco A in 50 mM phosphate pH 8.4. Spectra are representative of, at least, 3 different experiments. CID-MS/MS spectrum of the peptides containing modified Lys residues at: (A) Lys 3; (B) Lys 9; (C) Lys 30; (D) Lys 122; (E) Lys 128; and (F) Lys 136. This data is similar to Seco B, which is presented in the supporting information (Fig. S7). See Table S2 for more details concerning the tryptic digestion of SOD1.

**Fig. 8:**
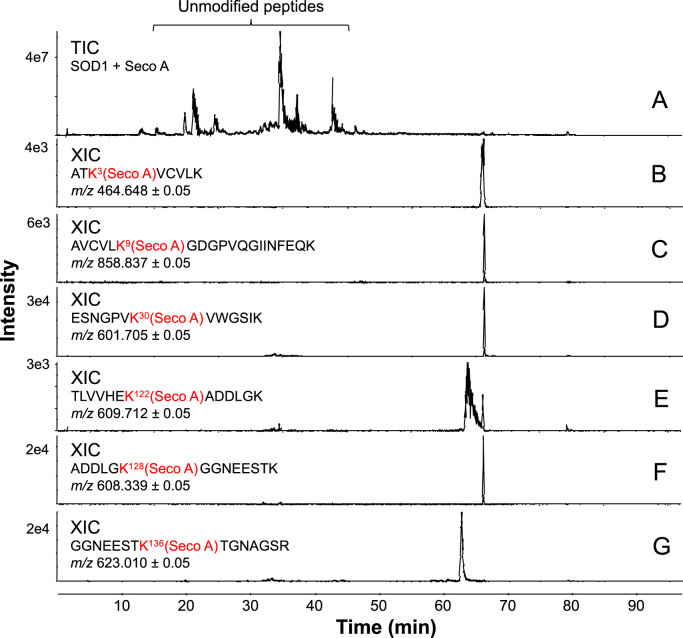
**Secosterol aldehyde adduction increases the retention time of the modified SOD1 peptides.** (A) Total ion chromatogram (TIC) of the tryptic digestion of SOD1 after incubation with Seco A. (B-G) Extracted ion chromatogram (XIC) of each modified peptide. Incubations contained 10 μM SOD1 with 250 μM Seco-A in 50 mM phosphate buffer at pH 8.4. Chromatograms are representative of, at least, 3 different experiments. This data is also representative to Seco B, which had identical result (Fig. S8). See Table S2 for more details concerning the tryptic digestion of SOD1.

Modified residues were found in the following peptides: 1) Lys 3 at the peptide ^1^ATK*AVC’VLK^9^; 2) Lys 9 at the peptide ^4^AVC’VLK*GDGPVQGIINFEQK^23^; 3) Lys 30 at the peptide ^24^ESNGPVK*VWGSIK^36^; 4) Lys 122 at the peptide ^116^TLVVHEK*ADDLGK^128^; 5) Lys 128 at the peptide ^123^ADDLGK*GGNEESTK^136^; and 6) Lys 136 at the peptide ^129^GGNEESTK*TGNAGSR^143^. All MS/MS analysis presented mass error below 5 ppm (Fig. 7 for Seco A; and Fig. S7 for Seco B; Table S1, Supporting Information). MS/MS spectra obtained for each modified peptide showed characteristic fragments indicative of secosterol-Lys adduction (Fig. 7 and Fig. S7). Of note, secosterol-adducted peptides eluted at longer retention times (60–70 min) when compared to the unmodified peptides that eluted between 20 and 50 min (Fig. 8). This chromatographic behavior can be explained by the hydrophobic nature of the aldehydes, which greatly increases the hydrophobicity of the modified peptide. Importantly, a similar increase in hydrophobicity would be expected for SOD1 adducted to secosterol aldehydes, thus enhancing its propensity to undergo aggregation. Quantification of SOD1 tryptic peptides adducted to Seco A or Seco B indicates that adduction occurs preferentially at Lys located at K136 >K122 >K128 =K30 =K3 >K9 (Fig. S8). Moreover, in agreement with the aggregation data (Fig. 3) modified peptides levels were higher for secoA compared to secoB.

## 4 Discussion

Neurodegenerative diseases are known to be associated with redox stress, which increases the production of lipid-derived electrophiles [47], [48]. Among the targets of these reactive products, proteins are likely the most suceptible to covalent modifications because their nucleophilic residues are easily attacked by aldehydes and other electrophiles [49], [50], [51]. Cholesterol 5,6-secosterol aldehydes (Seco A and Seco B) have been detected in brain tissues of patients with Lewy body [17] and Alzyheimer's disease [20]. These reactive oxysterols are reported to contribute to neurodegenerative disease pathology by inducing protein modification and aggregation [17], [18], [19], [20]. Here, we showed that secosterol aldehydes, mostly detected in the form of Seco B, are ubiquitously present in motor cortex, spinal cord and blood plasma of ALS rats. Relevantly, Seco B levels were found increased in plasma of symptomatic ALS rats compared to pre-symptomatic animals, indicating its potential association with disease progression. Also alterations in plasma may reflect the multisystemic character of the disease which affects multiple cell types (motor neurons, glia, muscle) and is likely to involve a non-cell autonomous toxicity [52], [53].

The transgenic ALS-SOD1^G93A^ rat model recapitulates many features of ALS, including motor neuron loss, mitochondrial dysfuction, astrogliosis, increased production of reactive oxygen species, vacuolization, and SOD1 aggregation [54]. Mechanisms by which mutant- and WT-SOD1 aggregates have been extensively studied [55], [56] but remain still elusive. Thus, having detected secosterol aldehydes in neuronal tissues, we asked the question whether they could induce SOD1 aggregation. Interestingly, secosterol aldehydes significantly enhanced WT-SOD1 aggregation of both apo- and holo-forms. As expected, aggregation was greater in the apo-SOD1 compared to holo, reflecting the poor stability of the metal-deficient form [27], [29]. Indeed, bioinformatic and structural biology studies have shown that apo-SOD1 in solution shows increased conformational dynamics and local unfolding [29], [57], that favors protein-protein, protein-ligand interactions, leading to protein aggregation.

*In vitro* studies have shown that apo-SOD1 aggregation takes days or weeks to occur [58]. Of note, our study showed that SOD1 aggregation was greatly accelerated in the presence of seco-A/B reaching a plateau at 12–24 h. This can be due to the hydrophobic nature of secosterols, which probably increases protein unfolding and aggregation. To better characterize the secosterol aldehyde-induced SOD1 aggregation, we decided to vary some reaction conditions, such as the pH and secosterol concentration. Remarkably, secosterol aldehyde-induced SOD1 aggregation was higher at pH above 7.4 (Fig. 3C). The pKa value of the thiol group is dependent on the structure of the enzyme and its local environment, but in general the pKa of thiols in peptides is usually around 9, whereas in proteins the values can be as low as 3.5 [59]. So, the increased aggregation observed at higher pH could be explained by the increase of thiolate levels on the protein, making it more prone to the formation of intermolecular disulfide cross-links under oxidative conditions. However, protein aggregation was not apparent when the same assay was performed with apo-SOD1 WT with cholesterol, even at high pH values (data not shown), showing that increased pH alone does not induce protein aggregation. The pH is not only important for thiol group reactivity, but also instrumental for the interaction between aldehyde and protein amino groups. Schiff base formation in proteins is favored at basic pH, that facilitates the nitrogen deprotonation from amino acids such as lysine, leading to exposure of their unpaired electrons [60]. Thus, our findings highlight the importance of pH to increase the Schiff base reaction between secosterol aldehydes and SOD1.

Secosterol aldehyde binding has been reported to increase aggregation of a number of disease-related proteins such as Aβ, apo B-100, α-synuclein and cytochrome c [15], [16], [18], [22]. In this context, it is suggested that secosterol aldehyde binding induce protein conformational changes, exposing non-polar domains or hydrophobic patches, which enhances their propensity to aggregation. In the case of SOD1, we initially hypothesized that this conformational change can also expose free cysteines that participates in aberrant intermolecular disulfide cross-linking and protein aggregation, favoring formation of β-amyloid type structures [32]. Data derived from the thioflavin T assay (Fig. 4A) confirmed exposition of hydrophobic domains, typically observed for amyloid fibrils. However, Congo red and TEM identified aggregates of amorphous nature (Fig. 4B and C), a morphology found in previous studies on SOD1 aggregation [32], [33], [34], [44]. More importantly, the aggregates observed here resemble those aggregates found in early aggregates found in animal and in vitro models [61], [62] and also in ALS patients [63], [64].

To characterize the secosterol covalent adduction to SOD1, we used two different strategies: the click chemistry assay (Fig. 5) and MALDI-TOF-MS analysis (Fig. 6). The first one has been used as an efficient tool for detecting adducts of lipid-derived electrophiles, including secosterol aldehydes, in proteins in vitro and in complex samples [40], [65], [66]. This assay allowed us to detect secosterol aldehyde-modified SOD1 species directly in SDS-PAGE gels. Using this approach, secosterol adduction was detected only in the high molecular weight region, with no apparent detection of adducts at SOD1 monomeric, dimeric or trimeric region. This data indicates that secosterol adduction generates species that readily undergo aggregation. Of note, MALDI-TOF analysis identified SOD1 species containing up to five simultaneous secosterol additions to the protein, greatly enhancing protein hydrophobicity and aggregation. The mass difference between the additions was 402 Da, consistent with secosterol adduction to Lys residues by Schiff base formation.

**Fig. 9:**
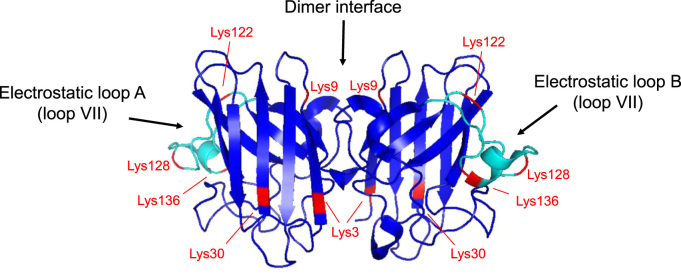
**Quaternary structure of SOD1 showing secosterol aldehyde (Seco A and Seco B) modified Lys residues.** Modified lysine residues are labeled in red and the electrostatic loops are labeled in cyan. The structure was drawn in PyMOL free software (www.pymol.org/). The PDB data used for illustration was the 3ECU - Crystal structure of human apo Cu, Zn Superoxide Dismutase (SOD1).

Adducted SOD1 was further digested and analyzed by nano-LC-MS/MS. We have identified modifications on six Lys residues (Lys 3, Lys 9, Lys 30, Lys 122, Lys 128 and Lys 136) out of the eleven residues in each SOD1 chain (Fig. 7). These residues are located in regions of SOD1 that have been described to be critical to aggregation, namely the electrostatic loop (loop VII; residues 120–143) and the dimer interface [67], [68] (Fig. 9). Loop VII in apo-SOD1 wild type and its mutants display the most dynamic structural alterations before global unfolding and aggregation [69], [70]. Importantly, the relative quantification of modified residues indicates that both Seco A and Seco B modifies Lys residues preferentially located at this loop (K136 >K122 >K128 =K30 =K3 >K9, Fig. S8). Thus, we hypothesize that secosterol adduction to Lys122, Lys128 and Lys136 greatly increases overall SOD1 hydrophobicity and aggregation. Secosterol adduction was also found at Lys3 and Lys9, located nearby the dimer interface. Based on the modified lysine locations, we can think of two scenarios for adduction. The Lys residues are covalently modified after monomerization or before monomerization. In the latter situation, we can expect that secosterol adduction will contribute to dimer disruption, increasing monomerization and aggregation.

Exposing hydrophobic surfaces is likely to be one of the critical factors involved in the formation and stability of SOD1 aggregates [32], [33], [34]. In our work, secosterol induced SOD1 aggregation was followed by increased hydrophobic surface, confirmed by the thioflavin T assay (Fig. 4A). This phenomenon could be simply explained by the intrinsic hydrophobic nature of the secosterols that dramatically increases the hydrophobicity of protein regions where they are covalently attached. The increase in hydrophobicity was clearly noticed for peptides bound to Seco A or Seco B that eluted at much later times in reversed-phase chromatography. In addition, secosterol adduction can induce protein unfolding leading to further exposition of protein hydrophobic patches. Together, the increased hydrophobicity associated with the exposition of hydrophobic residues can greatly contribute to SOD1 desestabilization and aggregation.

In conclusion, our data demonstrate that adduction of secosterol aldehydes to both apo- and holo-WT-SOD1 enhances protein aggregation. This effect added to the fact that these aldehydes are ubiquitously present in nervous tissue of ALS rats suggest that they may potentially contribute to protein aggregation mechanisms of the disease. Secosterol aldehydes modify Lys residues located preferentially at the eletrostatic loop and dimer interface. Adduction increases the overall protein hydrophobicity, leading to the disulfide-cross linked high molecular weight SOD1 aggregates. Taken together, our findings highlight the potential of reactive eletrophiles derived from cholesterol as inductors of SOD1 aggregation. Additional studies are necessary to evaluate the presence of SOD1-secosterol aldehyde adducts in vivo, as well as, to identify other critical protein targets, since this reactive eletrophile can modify any protein located nearby its site of formation. Furthermore, this work adds to evolving notion that secosterol aldehydes can have a significantly detrimental effect by their adduction to biomolecules.

## Footnotes

Appendix A Supplementary data associated with this article can be found in the online version at doi:10.1016/j.redox.2018.08.007.
