# Supplementary material for: Cholesterol secosterol aldehyde adduction and aggregation of Cu,Zn-superoxide dismutase: Potential implications in ALS
Source: Redox Biol. 2018 Aug 16;19:105–15. doi: 10.1016/j.redox.2018.08.007 (PMC6106709; doi:10.1016/j.redox.2018.08.007)
Supplement: Supplementary file 1 — Supplementary material [file mmc1.docx]

**Supplementary methods**

**Method S1: Synthesis of β-sitosterol 5,6-secosterol aldehyde (Sito-Ald) and its use as an internal standard for cholesterol secosterol aldehydes (Seco B) quantification**

Sitosterol 5,6-secosterol aldehyde (Sito-Ald) was synthesized by photooxidation following the same procedure described for Secosterol B (3β-hydroxy-5β-hydroxy-B-norcholestane-6β-carboxaldehyde) [8]. Briefly 200 mg of sitosterol was dissolved with 20 mL of chloroform and irradiated by two tungsten lamps (500 W) for 3 hours in the presence of 123 µM methylene blue as a photosensitizer. Sito-Ald was purified by flash column chromatography using 10 g of silica gel (230-400 mesh) using a hexane:ethyl acetate gradient from 95:5 to 40:60 (v/v). Aliquots were collected and checked by TLC analysis using isooctane:ethyl acetate (1:1, v/v) as eluent. Fractions containing pure Sito-Ald were collected, concentrated and checked by mass spectrometry and NMR.

**Figure S1.** Structures of β-Sitosterol and its derivative aldehyde (Sito-Ald).

**Method S2: Quantification of cholesterol and Seco B in cortex, spinal cortex and blood plasma**

With the purpose to get a precise quantification of cholesterol and Seco A/B in ALS rat tissues we added Sito-Ald as an internal standard. Briefly, an aliquot of 50 µL of 20 µM Sito-Ald was added to 200 µL of plasma or cortex and spinal cord homogenates before lipid extraction. Samples were then resuspended in 100 µL isopropanol before derivatization with 1-pyrenebutyric hydrazine (PBH) [43]. For the analysis an aliquot of 1 µL was injected into the HPLC equipped with Synergy C18 column (50 x 4.6 mm, 2.5 µm, Phenomenex). Separation was done using the following condition: 84% B, 0-5 min, 84-88% B 5-6 min, 88% B 6-12 min, 88-95 % B 12-13 min, 95 % 13-20 min, and 95-84% B 20-21 min. Fig. S2 shows a typical chromatogram for Seco A and Seco B quantification. Calibration curves was constructed by adding a fixed amount of Sito-Ald (5 µM, final concentration) into solutions containing 0.1, 0.25, 0.5, 1.0, 2.0, 3.0 and 5.0 µM Seco B. Curves were constructed by plotting Seco B concentrations *vs* are ratio of Seco B/Sito-Ald.

**Figure S2.** Chromatogram of 5,6-secosterol aldehyde quantification in lipid extracts of motor cortex, spinal cord and plasma from ALS rats. Sito-Ald (1 pmol) was added as the internal standard. After extraction and derivatization with the fluorescent probe 1-pyrenebutiric hydrazine (PBH), secosterol aldehydes were analyzed by HPLC-fluorescence dectector at Ex.:339 nm and Em.: 380 nm (details of the method can be found in Ref. 43).

**Recovery of Seco B in the samples**

To estimate the recovery of Seco B we calculated the recovery of its analogue, Sito-Ald (5 µM final concentration), that has been added into the plasma, motor cortex and spinal cord samples as the internal standard. We used as a reference the same amount of Sito-Ald (5 µM) that was added for the construction of standard curves. Mean recovery in motor cortex (CTRL 68.5±9.9, ALS 67.6±8.4) and spinal cord (CTRL 67.2±4.3, ALS 66.3±5.9) were lower compared to the plasma (CTRL 68.5±9.9, ALS 67.6±8.4) (Figure S3). Importantly, there were no differences between recoveries observed in all samples from control *vs* ALS groups.

**Figure S3.** Recovery of Sito-Ald added into plasma (A), motor cortex (B) and spinal cord (C) samples in control (n=10 animals) and ALS rats. Data represent the comparation between areas of Sito-Ald in samples and standard curve. There was no significant differences between groups (ANOVA, Tukey post-test).

**Precision of Seco B quantification**

To evaluate the precision of the method for Seco B quantification, we checked the reproducibility of three standard curves obtained in three different days and calculated the coefficient of variance (CV) as shown in Table S1. All points of the curves presented an inter-day CV lower than 6%.

**Table S1.** Precision and accuracy of three different standard curves for Seco B quantification constructed in three different days.

|  | Curve 1 | Curve 2 | Curve 3 |  |  |  |  |
| --- | --- | --- | --- | --- | --- | --- | --- |
| Seco B (pmol) | Area | Area | Area | Mean | SD | Precision  (CV %) | Accuracy  (RE %) |
| 0.5 | 2024402 | 1876999 | 1811857 | 1904419 | 108893 | 5.7 | -3.1 |
| 1 | 3569494 | 3654299 | 3281907 | 3501900 | 195181 | 5.6 | 1.6 |
| 2 | 6472759 | 6728139 | 6222007 | 6474302 | 253070 | 3.9 | 0.3 |
| 3 | 9175511 | 9984287 | 9162107 | 9440635 | 470864 | 5.0 | -0.2 |

The precision of the quantitation was expressed as percent coefficient of variance (CV %), calculated by dividing the standard deviation by the mean and then multiplied by 100. The accuracy of the analytic method was denoted by the relative error (RE %), calculated as percent of the mean deviation from the known amount, RE% = [(amount found by application of the constructed calibration curve – known amount) × 100 / (known amount)].

**Supplementary Figures**

**
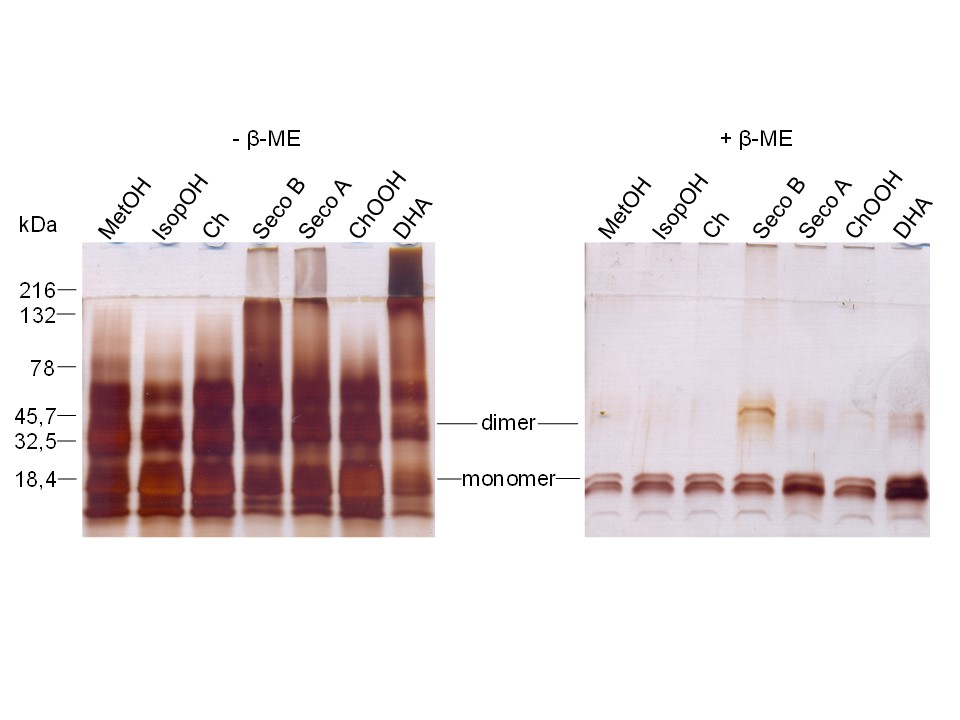
**

**Figure S4. SDS-PAGE of apo-SOD1 WT (10 μM) incubated in the presence of 250 μM of cholesterol, Seco A, Seco B, cholesterol hydroperoxides (ChOOH) or docosahexaenoic acid (DHA, 22:6 n-3) at 37 °C for 24 h.** Analysis was conducted under nonreducing (-β-ME) or reducing conditions (+β-ME). As a control we added the solvents used to solubilize the lipids (methanol, MetOH or isopropanol, IsopOH).

**
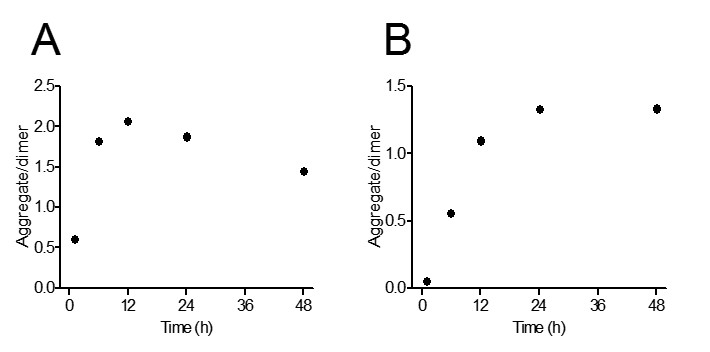
**

**Figure S5. Kinetics of apo-SOD1 aggregation monitored by size exclusion chromatography (SEC).** Apo-SOD1 WT (10 µM) was incubated in the presence of 250 µM secosterol A (A) and secosterol B (B) at 37 °C for 48h. Aliquots at 0, 1, 8, 12, 24 and 48 hours were analyzed by SEC with fluorescence detection. Aggregation was measured by the ratio between area of the aggregate and area of dimer.

**Figure S6.** Scheme of click chemistry assay.

**Figure S7.** MS/MS of the peptides resulting from the tryptic digestion of SOD1 after incubation with Seco B. Incubations contained 10 μM SOD1 with 250 μM Seco B in 50 mM phosphate pH 8.4. Spectra are representative of, at least, 3 different experiments. (A) MS/MS of the peptide referent to the Lys 3. (B) MS/MS of the peptide referent to the Lys 9. (C) MS/MS of the peptide referent to the Lys 30. (D) MS/MS of the peptide referent to the Lys 122. (E) MS/MS of the peptide referent to the Lys 128. (F) MS/MS of the peptide referent to the Lys 136. See Table S1 for more details concerning the tryptic digestion of SOD1.

**Figure S8.** Relative quantification of Seco A and Seco B modified SOD1 peptides. Incubations contained 10 μM SOD1 with 250 μM Seco B in 50 mM phosphate pH 8.4. Upper panel shows the quantification of adducted peptides obtained from three different incubations. The relative concentration of each modified peptide was calculated by the integration of the area under the XIC (extracted ion chromatogram) peak of the precursor ion using the Multiquant software and normalized against the total area of peptides. Lower panel shows the representative chromatograms of the modified peptides. * p<0.05, t-test.

**Table S2.** SecoA/B-modified peptides identified by LC-MS/MS after digestion with tripsin.

| Sequence | Residue | Unmodified (m/z) | Expected | Observed  (Seco-A/Seco-B) | Error (ppm) |
| --- | --- | --- | --- | --- | --- |
| ATK*AVC’VLK | Lys3 | 330.5319 (3+) | 464.6485 (3+) | 464.6475/464.6468 (3+) | -2.1/-3.6 |
| AVC’VLK*GDGPVQGIINFEQK | Lys9 | 724.7209 (3+) | 858.8375 (3+) | 858.8398/858.8399 (3+) | 2.6/2.7 |
| ESNGPVK*VWGSIK | Lys30 | 467.5893 (3+) | 601.7059 (3+) | 601.7057/601.7055  (3+) | -0.3/-0.6 |
| TLVVHEK*ADDLGK | Lys122 | 475.5963 (3+) | 609.7129 (3+) | 609.7144/609.7149 (3+) | 2.4/3.2 |
| ADDLGK*GGNEESTK | Lys128 | 474.2232 (3+) | 608.3398 (3+) | 608.3398/608.3400 (3+) | 0.0/0.3 |
| GGNEESTK*TGNAGSR | Lys136 | 488.8940 (3+) | 623.0106 (3+) | 623.0132/623.0129 (3+) | 4.1/3.6 |

*Aldehyde adduct; ’Carbamidomethyl adduct
